# Whole-Genome Sequence of Rickettsia parkeri Strain Atlantic Rainforest, Isolated from a Colombian Tick

**DOI:** 10.1128/MRA.00684-19

**Published:** 2019-09-26

**Authors:** Andrés F. Londoño, Nicole L. Mendell, Gustavo A. Valbuena, Andrew L. Routh, Thomas G. Wood, Steven G. Widen, Juan D. Rodas, David H. Walker, Donald H. Bouyer

**Affiliations:** a Department of Pathology, University of Texas Medical Branch, Galveston, Texas, USA; b Research Group on Veterinary Sciences Centauro, Facultad de Ciencias Agrarias, Universidad de Antioquia, Medellín, Colombia; c Berkeley School of Public Health, University of California, Berkeley, California, USA; d Department of Biochemistry and Molecular Biology, University of Texas Medical Branch, Galveston, Texas, USA; University of Maryland School of Medicine

## Abstract

Rickettsia parkeri is classified as a member of the alphaproteobacterial microorganisms, genus Rickettsia. Here, we report the complete genome sequence of Rickettsia parkeri strain Atlantic Rainforest, which was isolated from an Amblyomma ovale tick collected in the municipality of Necoclí, Colombia.

## ANNOUNCEMENT

Rickettsia parkeri, which is an obligately intracellular bacterium, has been reported across the American continents as infecting different tick species and has been associated with a relatively mild human disease (1). The full-length genome of the R. parkeri strain Atlantic Rainforest, isolated from a male *Amblyomma ovale* tick, is reported here. This tick was collected from a dog on 15 November 2010 in the municipality of Necoclí, Colombia (8°32.892′N, 76°34.429′W) (2).

The tick, code number Necoli_10_11, was homogenized with a Polytron system in 1 ml of sucrose-phosphate-glutamic acid (SPG) buffer (218 mM sucrose, 3.8 mM KH_2_PO_4_, 7.2 mM K_2_HPO_4_, and 3.9 mM glutamate monosodium [pH 7.0], filter sterilized), and the microorganism was isolated in Vero cells as described previously (2). The homogenate was passed through a 27-gauge needle, 200 μl was inoculated into a 6-well plate containing Vero cell monolayers, and the plate was incubated at 34°C in an atmosphere of 5% CO_2_ for 1 hour. Then, the cells were fed with medium containing 100 U/ml of penicillin, 100 μg/ml of streptomycin, and 0.25 μg/ml of amphotericin B. On day 3 postinoculation, the medium was changed to antibiotic/antimycotic-free medium, and this process was repeated once per week for 3 weeks. The cells were checked daily for cytopathic effect and once per week by immunofluorescence for detection of rickettsial antigen (2, 3). In week 3, when rickettsiae were detected, isolation was confirmed by PCR (2, 4). The sequences of *gltA*, *sca0*, and *sca5* (GenBank accession numbers KJ158742, KJ158741, and KJ158744) showed 100% identity with *R. parkeri* strain Atlantic Rainforest (2).

*R. parkeri* strain Atlantic Rainforest was cultivated in 20 150-cm^2^ cell culture flasks containing Vero cell monolayers and then released from the cells by sonication, pelleted by high-speed centrifugation, and purified by discontinuous renografin gradient ultracentrifugation (5, 6). DNA was extracted using the DNeasy blood and tissue kit (Qiagen, Hercules, CA). The manufacturer’s protocol was followed. The full genome was sequenced in parallel using MinION Nanopore and Illumina next-generation sequencing, and the consensus sequence was obtained as described below. For Illumina sequencing, 1 μg of genomic DNA was sheared to an average size of 500 bp using a Covaris S220 instrument. Illumina TruSeq libraries were prepared following the manufacturer’s instructions, and sequencing was performed on an Illumina MiniSeq instrument, using a high-output kit and paired-end 150-base reads; 23.4 million read pairs were obtained. Adapter sequences and low-quality base calls were removed with Trimmomatic version 0.22 using the following parameters: LEADING, 35; TRAILING, 35; SLIDINGWINDOW, 5:35; and MINLEN, 35 (7). For the Nanopore MinION run, 1 μg of input genomic DNA was sheared using a Covaris g-Tube (2 × 1 min at 1,500 relative centrifugal force [RCF]) to obtain DNA fragments of ∼8 kbp in length. DNA fragments were then prepared using Oxford Nanopore Technologies sequencing by ligation kit with native barcoding (SQK-LSK108 and NDB103) according to the manufacturer’s standard protocols. DNA libraries were loaded onto R9.4 FLO-MIN106 flow cells, and 1D sequencing was performed for a total of ∼24 h using the MinKNOW controller software until the data yield reduced substantially. Output FAST5 read data were demultiplexed and base called using Albacore version 2.1.10 with default settings, yielding 739,829 reads passing the filter. We used the MaSuRCa program version 3.2.4 and default parameters to assemble the genome with the combined Illumina and MinION reads. After assembly, both sets of reads were mapped to the new reference and used to correct 6 indels and 7 mismatches that were present in the MaSuRCA-assembled genome (8).

**FIG 1:**
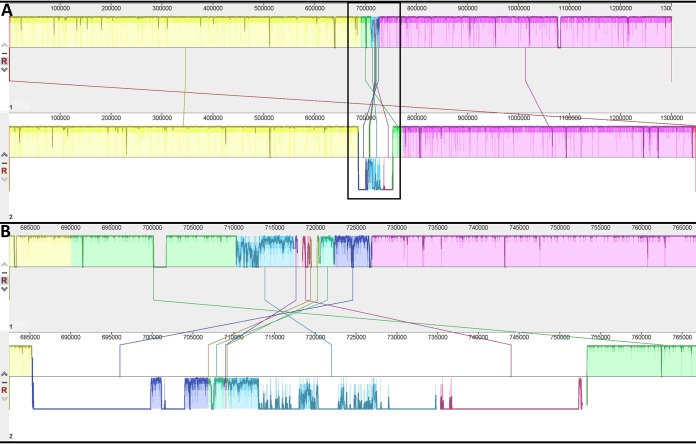
Alignment of the genomes of the *R. parkeri* Portsmouth (1) and Atlantic Rainforest (2) strains using the Mauve program (multiple-genome alignment). (A) Whole-genome alignment; (B) region with high changes (inset).

We used the Mauve program (multiple-genome alignment) to compare the genome with the genome of the type strain *R. parkeri* Portsmouth (9) (Fig. 1). In the whole genome, we identified several insertions, deletions, and inversion zones in comparison to the genome of the *R. parkeri* Portsmouth strain, including bacterial conjugation genes that included *traC*-like, *traB*-like, *traU*-like, and *traN*-like genes. The sequence has 1,348,030 bp, which is 47,644 bp longer than the *R. parkeri* Portsmouth strain genome.

### Data availability.

The genome sequence of Rickettsia parkeri strain Atlantic Rainforest, isolated from a Colombian tick, has been deposited in GenBank under the accession number CP040325. The accession numbers for the raw MinION and Illumina reads are SRX6651593 and SRX6651594, respectively.
